# Virtual Bronchoscopic Pathfinder (VBP): An Open-Source Web-Based System for Airway Segmentation, Cost-Field Path Planning, and Cross-Device 3D Navigation

**DOI:** 10.3390/tomography12070095

**Published:** 2026-06-29

**Authors:** Young Kim, Sunggyu Choi, Chulmin Park, Woojin Park, Doohee Lee

**Affiliations:** 1 Department of Computer Science and Engineering, Seoul National University, 1 Gwanak-ro, Gwanak-gu, Seoul 08826, Republic of Korea; 2 Department of Computer Science Engineering, College of IT, Kangwon National University, Chuncheon 24341, Republic of Korea; 3 Growth Strategy Division, DOUZONEBIZON Co., Ltd., Chuncheon 24465, Republic of Korea; 4 Department of Research and Development, ZIOVISION Co., Ltd., Chuncheon 24341, Republic of Korea; 5 Department of Data Science, Graduate School, Kangwon National University, Chuncheon 24341, Republic of Korea

**Keywords:** Virtual Bronchoscopic Pathfinder (VBP), Virtual Bronchoscopic Navigation (VBN), pulmonary airway segmentation, topology-preserving skeletonization, bidirectional Dijkstra algorithm, vtk.js, lung cancer

## Abstract

**Background/Objectives:** Virtual Bronchoscopic Navigation is used to guide bronchoscopes toward peripheral pulmonary lesions, but broad clinical and research adoption remains limited by the cost of proprietary software and by segmentation failures in small distal airways that can interrupt path planning. This study presents Virtual Bronchoscopic Pathfinder, an open-source, web-based system designed to provide automated airway segmentation, robust path generation, and browser-based three-dimensional visualization. **Methods**: The system integrates five components: a connectivity-aware deep learning model for pulmonary airway segmentation using Connectivity-Aware Surrogate and Local-Sensitive Distance modules; TotalSegmentator for automated tumor localization; a topology-preserving three-dimensional thinning algorithm implemented in C++ for centerline extraction; a bidirectional Dijkstra algorithm operating on a three-tier anatomical cost field with centerline, airway lumen, and parenchymal costs; and a zero-footprint visualization interface built on vtk.js with synchronized axial viewing and interactive volume rendering. VBP was validated on 306 thin-section CT series from 154 subjects in the public Lung-PET-CT-Dx dataset. **Results:** Among the 306 CT series, 33 series (10.8%) were excluded because of scanner-specific segmentation artifacts. In the remaining 273 anatomically valid series, the system successfully generated complete end-to-end navigation paths for all cases. The overall pipeline success rate was therefore 273 of 306 series (89.2%). The web-based interface was also confirmed to operate without client-side installation across desktop, laptop, and mobile device configurations. **Conclusions:** Virtual Bronchoscopic Pathfinder demonstrates that a reliable and accessible virtual bronchoscopic navigation workflow can be constructed entirely from open-source components. By combining connectivity-aware segmentation, cost-field path planning, and browser-based visualization, the system provides a practical foundation for imaging informatics research and future development of intra-procedural bronchoscopic guidance.

## Simple Summary

Peripheral lung lesions are difficult to reach with conventional bronchoscopy because the airway branches become narrow and complex. This study presents Virtual Bronchoscopic Pathfinder, a web-based system that automatically analyzes chest CT images, identifies airway routes, and displays a three-dimensional navigation path to support bronchoscopic planning. The system was tested using a public lung cancer imaging dataset and successfully generated navigation paths in most anatomically valid CT series. By combining automated image analysis with browser-based visualization, this approach may help make virtual bronchoscopy planning more accessible for research, education, and future clinical workflow development.

## 1. Introduction

The diagnosis and treatment of peripheral pulmonary lesions (PPLs) represent a critical challenge in respiratory medicine, requiring precise navigation through the complex bronchial tree [1,2]. Virtual Bronchoscopic Navigation (VBN) addresses this challenge by reconstructing three-dimensional airway structures from Computed Tomography (CT) images, thereby providing a pre-procedural roadmap for guiding flexible bronchoscopes toward peripheral targets [3]. However, a persistent limitation of existing deep-learning-based segmentation models is the breakage of small distal bronchi, which disrupts topological continuity and compromises path-planning reliability [4].

Prior work has addressed individual components of the VBN workflow in isolation. Benchmarking efforts such as EXACT’09 [5] and ATM’22 [6] identified topological continuity failures in peripheral bronchi as a persistent challenge. Deep learning segmentation methods [7,8,9,10] have improved peripheral bronchiole detection but do not provide end-to-end navigation pipelines. Existing open-source VBN tools [11,12,13] require local installation or proprietary hardware and lack connectivity-aware segmentation. Clinical path-planning studies [14,15] demonstrate feasibility but rely on closed commercial stacks. No existing system unifies connectivity-aware segmentation, skeleton-breakage-resilient path planning, and zero-footprint web-based visualization into a single publicly deployable pipeline.

In this study, we present **Virtual Bronchoscopic Pathfinder (VBP)**, a complete, open-source, web-based VBN system accessible at https://vbn.ziovision.ai (accessed on 21 February 2026). The contributions of VBP are as follows: (i) automated airway segmentation using a connectivity-aware deep learning model [4]; (ii) automated tumor localization via TotalSegmentator [16]; (iii) high-performance C++ topology-preserving skeletonization [17,18]; (iv) multi-target bidirectional Dijkstra-based path planning with an anatomically hierarchical cost field [19]; and (v) a zero-footprint web-based visualization interface built on the vtk.js engine [20,21]. The pipeline was validated on 306 thin-section CT series from 154 subjects in the Lung-PET-CT-Dx dataset [22].

## 2. Materials and Methods

**Figure 1:**
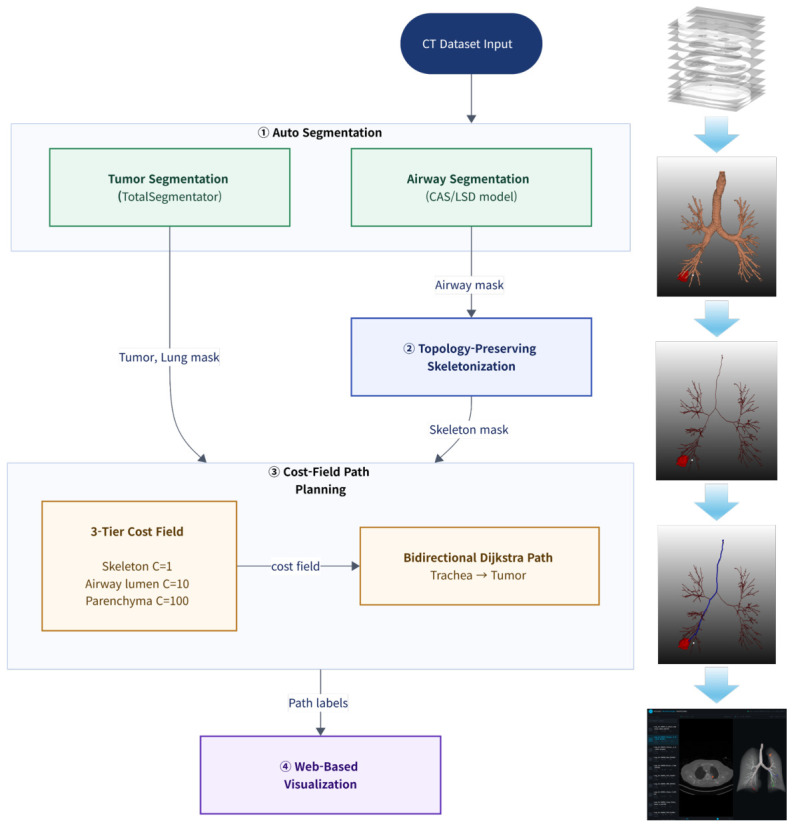
Overview of the Virtual Bronchoscopic Pathfinder (VBP) pipeline. The left column shows the four processing stages and their data flow; the right column shows representative outputs at each stage.

**Figure 2:**
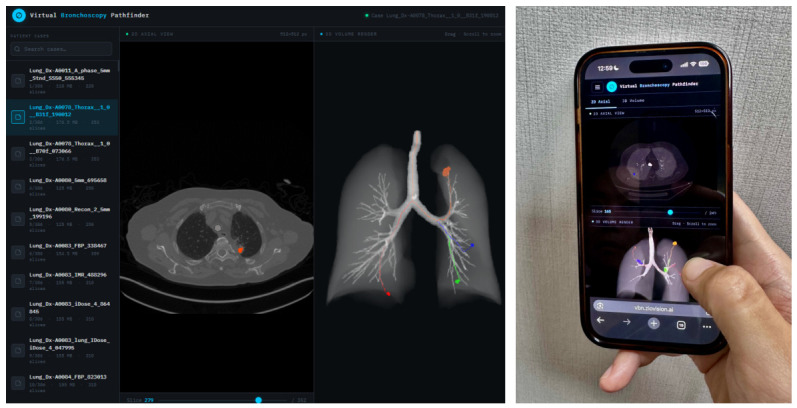
The VBP visualization system (https://vbn.ziovision.ai, accessed on 21 February 2026) across device form factors.

An overview of the VBP pipeline is shown in Figure 1. The system comprises four sequential stages executed on a shared file system: (i) automated airway and tumor segmentation from raw CT data; (ii) topology-preserving 3D skeletonization of the airway lumen mask; (iii) cost-field-based multi-target path planning via bidirectional Dijkstra search; and (iv) zero-footprint web-based 3D visualization. The right column of Figure 2 illustrates the representative output at each stage, from the raw axial CT stack through progressively refined anatomical structures to the final navigation path rendered in the browser interface. Each stage is described in detail in Section 2.1, Section 2.2, Section 2.3, Section 2.4 and Section 2.5.

### 2.1. Dataset

We utilized the **Lung-PET-CT-Dx** dataset, a large-scale public repository of pathologically verified lung cancer cases provided by The Cancer Imaging Archive (TCIA) [22]. The dataset was downloaded on 22 December 2025. Although no publicly available dataset provides bronchoscopy-specific ground truth (e.g., per-generation airway labels or procedural navigability annotations), Lung-PET-CT-Dx offers the largest available cohort of real lung cancer CT data with confirmed pathological diagnoses, making it the most appropriate resource for large-scale feasibility validation of a VBN pipeline. The dataset encompasses three histological subtypes coded in the subject identifier prefix—adenocarcinoma (*Lung_Dx-A*, n=116), large-cell carcinoma (*Lung_Dx-B*, n=9), and squamous cell carcinoma (*Lung_Dx-G*, n=29)—yielding 154 unique subjects in total. CT acquisitions were performed between 2009 and 2011 using scanners from three manufacturers: Siemens (82.0%), Philips (12.1%), and GE Medical Systems (5.9%).

To ensure the spatial resolution required for peripheral airway reconstruction, we applied the following inclusion criteria. Only CT series containing **200 or more axial slices** were retained, as this threshold reliably identifies sub-millimeter acquisitions (typically 1.0 mm slice thickness) that preserve the structural continuity of distal bronchi [4]. In addition, only structural CT data stored in 16-bit grayscale format were included; multimodal fused series containing PET information were excluded. These criteria yielded a final cohort of **306 series from 154 subjects** (totaling approximately 54.6 GB and 105,931 axial images). The key characteristics of the retained series are summarized in Table 1.

Regarding tumor localization, we did not use the XML bounding-box annotations supplied with the dataset [22]. Instead, we performed independent voxel-wise tumor segmentation using TotalSegmentator [16], as described in Section 2.2, to obtain the precise 3D masks required for center-of-mass (COM) computation in the path-planning stage. Of the 306 retained series processed through VBP, 33 (10.8%) produced structurally implausible airway masks attributable to scanner-specific acquisition artifacts; these cases are analyzed in detail in Section 4. It should be noted that no automated thoracic field-of-view (FOV) restriction was applied in the current preprocessing pipeline; all retained series, including whole-body acquisitions, were passed to the airway segmentation model in their entirety. The Type 3 failure case documented in Section 4 directly demonstrates the consequences of this omission, and the incorporation of a thoracic region-of-interest extraction step as a preprocessing safeguard is identified as a near-term improvement for future pipeline iterations.

### 2.2. Automated Segmentation

Segmentation of the pulmonary airway and tumor was performed using two independent deep learning frameworks.

- **Airway Segmentation:** We employed a connectivity-aware model incorporating the **Connectivity-Aware Surrogate (CAS)** and **Local-Sensitive Distance (LSD)** training modules [4]. These modules explicitly penalize topological breakage during training, ensuring that the resulting binary mask maintains the structural continuity of distal bronchi—a prerequisite for generating uninterrupted navigation paths.
- **Tumor Segmentation:** Automated voxel-wise segmentation was performed using **TotalSegmentator** [16], an open-source framework trained to delineate 104 anatomical structures, including pulmonary masses and nodules. The resulting 3D tumor mask was used to compute the target center of mass (COM), which serves as the endpoint for the path-planning algorithm.

### 2.3. Airway Skeletonization

The segmented airway mask was reduced to a 1-voxel-thickness medial centerline using a **topology-preserving 3D thinning algorithm** [17], implemented in C++ and ported from the Java-based Skeletonize3D plugin (version 2.1.1, accessed on 12 August 2025) of the ImageJ/Fiji framework (ImageJ version 1.54p, accessed on 17 February 2025) [18]. This deterministic, parameter-free implementation was chosen for its reproducibility and established validation in 3D medical image analysis. The algorithm iteratively removes border voxels from the binary mask while preserving three topological properties: **endpoint voxels**, **Euler invariance**(verified via a 256-entry LUT), and **simple-point** status (via octree-based connectivity analysis). The complete procedure is formalized in Algorithm 1.
**Algorithm 1** Topology-Preserving 3D Thinning**Require:** 
Binary airway mask *I* of size W×H×D**Ensure:** 
Skeleton image *O* (1-voxel-thick medial centerline)  1:O← copy of *I* (binarized to {0,1})  2:Pre-compute Euler LUT E[256]                   ▷ fillEulerLUT  3:unchangedBorders←0  4:**while** unchangedBorders<6 **do**  5:    unchangedBorders←0  6:    **for** d∈{N,S,E,W,U,B} **do**                     ▷ 6 border directions  7:        candidates←∅; noChange←true  8:        **for** each foreground voxel v=(x,y,z) in *O* **do**  9:           **if** *v* is not a border voxel in direction *d* **then continue**10:           **end if**11:           **if** isEndPoint(O,v) **then continue**12:           **end if**                         ▷≤1 foreground neighbor13:           N← 27-neighborhood of *v* in *O*14:           **if** not isEulerInvariant(N,E) **then continue**15:           **end if**16:           **if** not isSimplePoint(N) **then continue**17:           **end if**                            ▷ octree connectivity18:           candidates.push(v)19:        **end for**20:        **for** each v∈candidates **do**21:           Re-fetch N← 27-neighborhood of *v* in *O*              ▷ concurrent safety22:           **if** isSimplePoint(N) **then**23:               O[v]←0; noChange←false24:           **end if**25:        **end for**26:        **if** noChange **then** unchangedBorders +=127:        **end if**28:    **end for**29:**end while**30:**return** *O*

### 2.4. Path Planning

The path-planning stage takes the skeleton *S* produced by Section 2.3, the airway mask $M_{\mathrm{air}}$, and up to 25 tumor masks ${\left\{M_{k}\right\}}_{k=1}^{K}$ as input, and outputs a single integrated NIfTI volume encoding all navigation paths. The stage is organized into three sub-steps: cost field construction, multi-target path finding, and output labeling.

#### 2.4.1. Cost Field Construction

Rather than treating path planning as a pure graph traversal on the skeleton alone, we construct a continuous **3D cost field** $C:V\to R_{>0}$ over the entire CT volume *V* so that the planner can bridge local skeleton discontinuities. Each voxel *v* is assigned a traversal cost according to its anatomical classification:

- **Skeleton voxel** (v∈S): C(v)=1.0—the planner preferentially follows the bronchial medial axis.
- **Airway lumen** ($v\in M_{\mathrm{air}}\setminus S$): C(v)=10.0—a low-penalty fallback that keeps the path within the airway when skeleton gaps exist.
- **Lung parenchyma/tissue** (otherwise): C(v)=100.0—a high-penalty region traversed only when the target lies beyond the segmentable distal bronchi.

This three-tier weighting guarantees that any reachable tumor COM always has a valid path from the trachea while enforcing a strong preference for anatomically meaningful routes. The construction procedure is formalized in Algorithm 2.
**Algorithm 2** Cost-Field Construction for VBP Path Planning**Require:** 
Skeleton *S*, Airway mask $M_{\mathrm{air}}$, Volume *V* of size W×H×D**Ensure:** 
Cost field C[0…|V|−1] of type float32  1:**for** each voxel index i∈[0,|V|) **do**  2:    **if** S[i]=1 **then**  3:        C[i]←1.0                            ▷ Skeleton centerline  4:    **else if** $M_{\mathrm{air}}\left[i\right]=1$ **then**  5:        C[i]←10.0                          ▷ Airway lumen fallback  6:    **else**  7:        C[i]←100.0                        ▷ Lung parenchyma/tissue  8:    **end if**  9:**end for**10:**return** *C*

#### 2.4.2. Multi-Target Bidirectional Dijkstra Path Finding

The core path-finding engine is a **bidirectional Dijkstra algorithm** [19] adapted for 3D voxel grids with 26-neighbor connectivity, derived from the open-source dijkstra3d library [23]. Simultaneous forward-and-reverse expansion reduces the effective search radius from O(|V|) to $O(\sqrt{\left|V\right|})$, providing significant speedups over unidirectional search on large CT volumes.

The **source** $v_{\mathrm{src}}$ is automatically identified as the skeleton voxel with the maximum *Z*-coordinate, corresponding to the superior margin of the trachea. For a patient case containing *K* tumors (K≤25), the same cost field *C* is reused for all *K* path-finding calls, eliminating redundant computation. Each target $v_{k}=\mathrm{COM}\left(M_{k}\right)$ is the center of mass of the *k*-th tumor mask. The complete multi-target path-finding procedure is formalized in Algorithm 3.
**Algorithm 3** Multi-Target Bidirectional Dijkstra Path Finding**Require:** 
Cost field *C*, Skeleton *S*, Tumor masks ${\left\{M_{k}\right\}}_{k=1}^{K}$, K≤25**Ensure:** 
Output label volume *L* (UINT8), initialized to $M_{\mathrm{air}}$ label 2  1:$v_{\mathrm{src}}\leftarrow \arg\max_{v\in S}\;Z\left(v\right)$                    ▷ Superior tracheal voxel  2:L← encode *S* as label 3, $M_{\mathrm{air}}$ as label 2, background as 1  3:**for** k=1 **to** *K* **do**  4:    $v_{k}\leftarrow \mathrm{round}\left(\mathrm{COM}(M_{k})\right)$              ▷ Target: tumor center of mass  5:    *//—Bidirectional Dijkstra—*  6:    ${\mathrm{dist}}_{\mathrm{fwd}}\left[v\right]\leftarrow +\infty \;\forall v$; ${\mathrm{dist}}_{\mathrm{fwd}}\left[v_{\mathrm{src}}\right]\leftarrow 0$  7:    ${\mathrm{dist}}_{\mathrm{rev}}\left[v\right]\leftarrow +\infty \;\forall v$; ${\mathrm{dist}}_{\mathrm{rev}}\left[v_{k}\right]\leftarrow 0$  8:    $Q_{\mathrm{fwd}}.\mathrm{push}\left(0,\;v_{\mathrm{src}}\right)$; $Q_{\mathrm{rev}}.\mathrm{push}\left(0,\;v_{k}\right)$  9:    ${\mathrm{cost}}^{*}\leftarrow +\infty$; $v_{\mathrm{meet}}\leftarrow \mathrm{null}$10:    forward←false11:    **while** $Q_{\mathrm{fwd}}\neq$ ∅ **and** $Q_{\mathrm{rev}}\neq$ ∅ **do**12:        forward←¬forward                ▷ Alternate between queues13:        **if** forward **then**14:           $u\leftarrow Q_{\mathrm{fwd}}.\mathrm{pop}\_\min\left(\right)$15:           **if** ${\mathrm{dist}}_{\mathrm{rev}}\left[u\right]<+\infty$ **then**16:               $c\leftarrow |{\mathrm{dist}}_{\mathrm{fwd}}\left[u\right]|+|{\mathrm{dist}}_{\mathrm{rev}}\left[u\right]|+C\left[v_{k}\right]-C\left[u\right]$17:               **if** $c<{\mathrm{cost}}^{*}$ **then** ${\mathrm{cost}}^{*}\leftarrow c$; $v_{\mathrm{meet}}\leftarrow u$18:               **else break**19:               **end if**20:           **end if**21:           Relax 26-neighbors of *u* in ${\mathrm{dist}}_{\mathrm{fwd}}$, $Q_{\mathrm{fwd}}$, ${\mathrm{parents}}_{\mathrm{fwd}}$22:        **else**23:           $u\leftarrow Q_{\mathrm{rev}}.\mathrm{pop}\_\min\left(\right)$24:           **if** ${\mathrm{dist}}_{\mathrm{fwd}}\left[u\right]<+\infty$ **then**25:               $c\leftarrow |{\mathrm{dist}}_{\mathrm{fwd}}\left[u\right]|+|{\mathrm{dist}}_{\mathrm{rev}}\left[u\right]|+C\left[v_{k}\right]-C\left[u\right]$26:               **if** $c<{\mathrm{cost}}^{*}$ **then** ${\mathrm{cost}}^{*}\leftarrow c$; $v_{\mathrm{meet}}\leftarrow u$27:               **else break**28:               **end if**29:           **end if**30:           Relax 26-neighbors of *u* in ${\mathrm{dist}}_{\mathrm{rev}}$, $Q_{\mathrm{rev}}$, ${\mathrm{parents}}_{\mathrm{rev}}$31:        **end if**32:    **end while**33:    *//—Path Reconstruction & Labeling—*34:    **if** $v_{\mathrm{meet}}\neq \mathrm{null}$ **then**35:        $P_{k}\leftarrow$ backtrack from $v_{\mathrm{meet}}$ via ${\mathrm{parents}}_{\mathrm{fwd}}$ and ${\mathrm{parents}}_{\mathrm{rev}}$36:        **for** each $v\in P_{k}$ **do**37:           L[v]←10k+2                        ▷ Path#k label38:        **end for**39:        $L[v_{k}]\leftarrow 10k+1$                         ▷ Tumor#k label40:    **end if**41:**end for**42:**return** *L*

#### 2.4.3. Output Label Schema

The final output is stored as a NIfTI file using the following label encoding, which is shared between the C++ backend and the web visualization system:

- Label **1**: Lung mask (background context)
- Label **2**: Airway lumen
- Label **3**: Skeletonized centerline tree
- Label 10k+1 (k=1,…,25): Tumor *k*
- Label 10k+2 (k=1,…,25): Navigation path to Tumor *k*

### 2.5. Web-Based Visualization System

The complete VBP pipeline executes five sequential stages as independent modules: (1) CT filtering and NIfTI conversion; (2) airway and tumor segmentation; (3) C++ topology-preserving skeletonization; (4) multi-target Dijkstra path planning; and (5) web-based visualization. The final stage delivers results through a **zero-footprint** web-based interface accessible at https://vbn.ziovision.ai (accessed on 21 February 2026), following design principles established in prior web-based medical imaging platforms [20,21].

#### 2.5.1. Backend Architecture

The backend is implemented as a **Flask** (Python) application exposing a RESTful API.

For **2D axial viewing**, the server asynchronously processes CT and mask NIfTI volumes upon case selection.

For **3D volume rendering**, a separate endpoint returns the complete mask volume as a raw uint8 byte stream encoded in Base64, allowing the client to reconstruct the full vtkImageData object in one operation.

#### 2.5.2. Frontend Interface

The frontend is a responsive single-page application (SPA) that adapts to the available viewport. On **desktop and laptop** displays, the interface presents a **dual-panel layout** with an interactive 2D axial viewer and a 3D volume renderer powered by **vtk.js**. On **mobile devices**, the layout switches to a vertically stacked single-column arrangement with touch-optimized controls, enabling point-of-care review from a smartphone or tablet.

Both views are illustrated in Figure 2.

## 3. Results

### 3.1. Quantitative Evaluation

VBP was validated on all 306 thin-section CT series (154 subjects) from the Lung-PET-CT-Dx dataset [22]. Pipeline-level outcomes are summarized in Table 2.

Of the 306 series, 33 (10.8%) were excluded from path planning due to structurally implausible airway masks arising from scanner-specific acquisition artifacts (detailed in Section 4). For the remaining 273 series (89.2%), the bidirectional Dijkstra algorithm successfully generated a continuous path from the proximal trachea to the center of mass (COM) of every TotalSegmentator-detected tumor, yielding a path-generation success rate of 100% within the anatomically valid subset.

### 3.2. Qualitative Assessment

Qualitative review confirmed that generated trajectories were anatomically plausible in representative cases. The computed paths followed the bronchial midline through distal branches reconstructed by the connectivity-aware segmentation model [4] and correctly transitioned to the airway–lumen cost layer where minor skeleton discontinuities were present. In cases where tumors were located beyond the extent of the segmentable bronchi, the planner successfully traversed the parenchyma cost layer to reach the target COM, as designed by the three-tier cost field.

### 3.3. Web-Based System: Cross-Device Operability

The VBP visualization system described in Section 2.5 was evaluated as a proof-of-concept for zero-footprint deployment across three device classes: desktop workstation, laptop, and mobile smartphone. The objective was to confirm basic cross-device operability rather than to conduct a formal usability study. Server-side NIfTI preprocessing and slice encoding were completed without observable latency issues on the test server, after which all 2D slices and the 3D mask volume were resident in the client browser without further server calls. The 3D volume renderer produced smooth interactive rotation and zoom on desktop and laptop hardware. On mobile devices, the responsive layout and touch controls functioned correctly, confirming that point-of-care review is feasible without application installation. Operation was verified in current releases of Chrome, Firefox, and Safari. A formal evaluation of clinical usability, including task completion rates and user experience metrics, is identified as a priority for future work.

## 4. Discussion

Beyond software integration, the scientific contribution of VBP lies in its ability to transform CT-derived airway segmentations into anatomically consistent graph representations suitable for navigation analysis. The framework preserves airway topology across segmentation, skeletonization, graph abstraction, and path-planning stages, thereby addressing a clinically important but often underemphasized aspect of CT image analysis. Each component has been validated in isolation in prior literature [4,16,17,19], but no prior work has assembled them into a deployable, publicly accessible VBN pipeline. The engineering challenge of reliable integration—ensuring consistent coordinate spaces, label conventions, and data handoffs across heterogeneous tools—is non-trivial, and the result is a system that enables large-scale informatics studies that would be practically infeasible with existing commercial or desktop-only alternatives. While conventional VBN systems often depend on proprietary software licenses and dedicated workstations, VBP demonstrates that equivalent end-to-end functionality can be achieved using exclusively open-source tools and a public dataset [16,22], lowering the barrier to adoption for research institutions worldwide.

It is important to clarify that the evaluation presented in this study was designed as an end-to-end system validation rather than a component-level algorithmic benchmark. Because VBP integrates multiple heterogeneous modules—including airway segmentation, skeletonization, path planning, and web-based visualization—our primary evaluation objective was to assess the robustness and operational feasibility of the complete workflow across a large multi-center CT cohort. This system-oriented evaluation perspective is consistent with established frameworks for assessing integrated AI pipelines in medical imaging [24,25] and forms the basis for future workflow-level validation studies that will incorporate clinical performance metrics and prospective radiology review.

A technically novel aspect of VBP is the three-tier anatomical cost field. Prior path-planning approaches for bronchoscopic navigation operate exclusively on the skeleton graph and therefore fail whenever a skeleton discontinuity interrupts the route [12,14]. By constructing a dense volumetric cost field that assigns graded traversal penalties to skeleton voxels, airway–lumen voxels, and parenchymal voxels, VBP guarantees that a continuous path from the trachea to any reachable tumor COM always exists, regardless of local skeleton breakage. This graceful degradation property—preferring skeleton routes but falling back through the lumen and, if necessary, parenchyma—directly addresses the fundamental fragility of skeleton-only planners in the presence of segmentation imperfections [4]. Furthermore, the cost field is constructed only once per patient case and reused for all *K* tumor targets (K≤25), making the multi-target extension computationally efficient.

**Figure 3:**
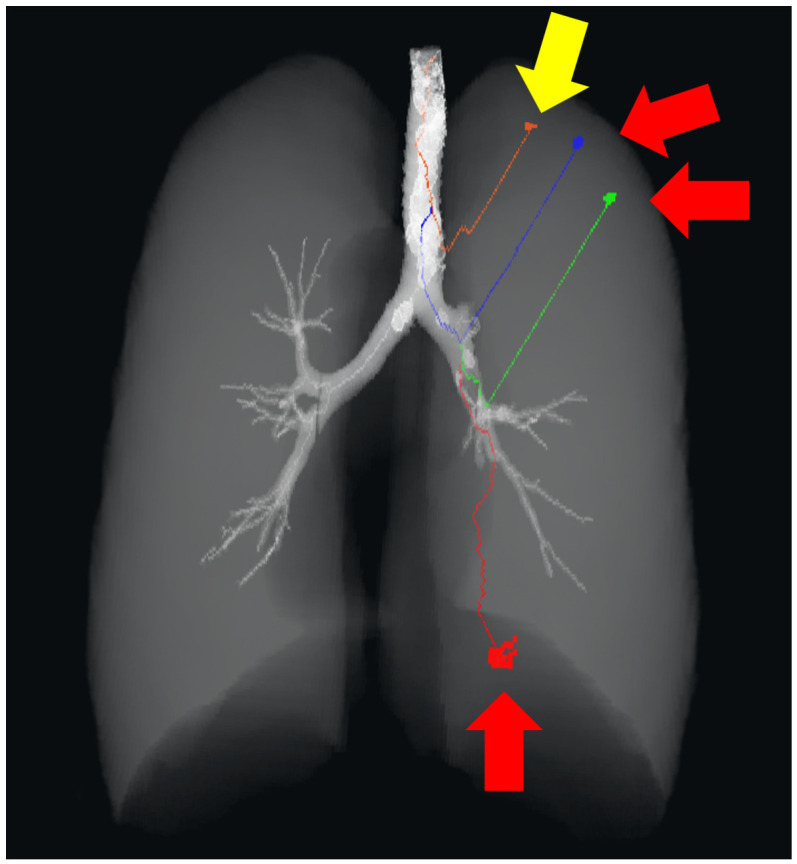
Representative path-planning failure cases of VBP (case *Lung_Dx-A0110_5mm_197561*). (**Yellow arrow**) A tumor whose center of mass lies in immediate proximity to the trachea causes the bidirectional Dijkstra algorithm to select a direct path from the tracheal centerline to the target, bypassing normal bronchial routing and yielding a clinically non-navigable trajectory. (**Red arrows**) Tumors located at or beyond the peripheral boundary of the lung, where no segmentable distal bronchi are present, force the planner to traverse an extended parenchymal cost layer. The resulting paths are geometrically valid but anatomically implausible, spanning an excessive distance through lung tissue rather than following a bronchoscopic route.

Several limitations of the current system should be acknowledged. Excluding the 33 cases with scanner-artifact segmentation failures analyzed below, the remaining 273 anatomically valid series are subject to two additional path-planning failure modes, both illustrated in Figure 3. Although the precise prevalence of each mode was not quantified in this study—an acknowledged limitation—representative examples are presented to characterize their mechanisms. **Trachea-proximal path shortcuts** occur when a tumor COM lies near the trachea or a main bronchus, causing the planner to select a direct, anatomically non-navigable trajectory rather than descending through the bronchial tree; this reflects the cost field’s lack of anatomical depth encoding. **Excessive parenchymal traversal** occurs when tumors lie beyond the extent of segmentable distal bronchi, forcing the planner into the high-cost parenchymal layer for an extended distance. A further limitation is that TotalSegmentator-derived tumor COM coordinates were not independently validated against radiologist-confirmed lesion centroids; segmentation confusion with adjacent vessels or consolidated parenchyma may displace the COM and misdirect navigation paths. Additionally, the tracheal source identification heuristic (maximum *Z*-coordinate of the skeleton) may be unreliable in heavily tilted or whole-body acquisitions, and no automated thoracic field-of-view restriction was applied, contributing to the scanner-artifact failures described below.

Future work will address these limitations through four directions: (1) introduction of a CT-anatomy-aware pre-filter to exclude non-airway structures before skeletonization; (2) addition of a minimum bronchial generation depth constraint to prevent anatomically implausible proximal shortcuts; (3) a prospective study with independent thoracic radiology review to formally quantify path-planning failure prevalence and assess clinical navigability; and (4) extension of VBP toward real-time intra-procedural guidance and low-latency multi-user collaboration.

**Figure 4:**
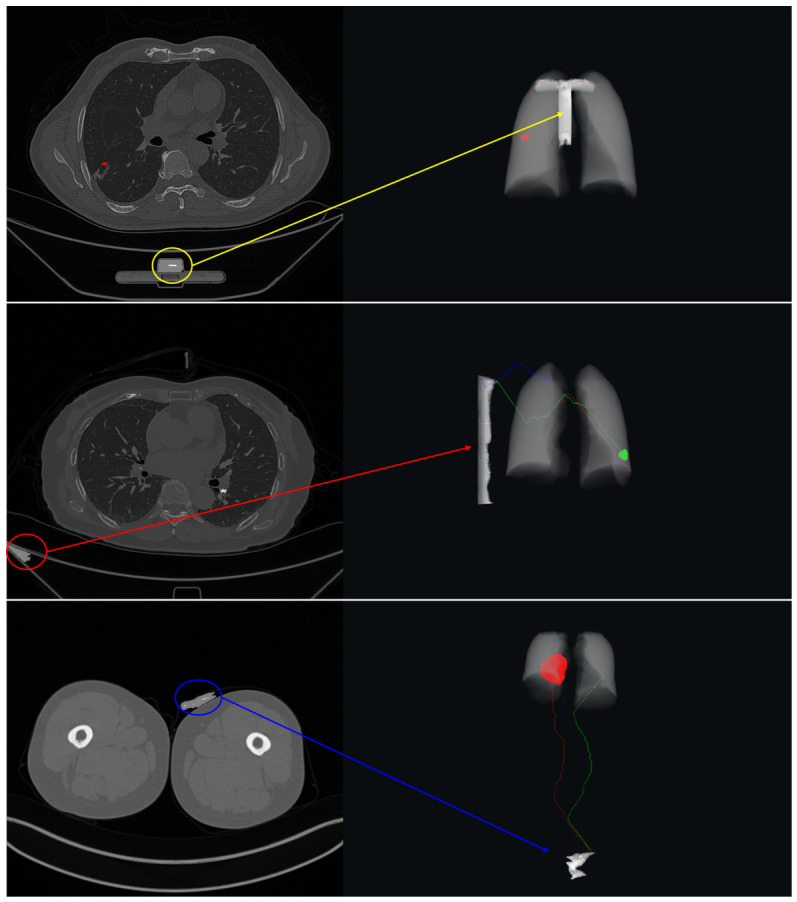
Three types of airway segmentation failure observed in the Lung-PET-CT-Dx dataset. For each row, the axial CT slice (left) and the resulting 3D path-planning output (right) are shown. (**Top, yellow**) Type 1: the central longitudinal air channel of a Philips scanner bed is misclassified as the trachea (case *Dx-A0083_FBP_338467*; Philips, *Chest 3D IMR*). (**Middle, red**) Type 2: the tapered lateral edge of the scanner bed is misclassified as a bronchial segment (case *Dx-A0084_IMR_575314*; Philips, *Chest 3D IMR*). (**Bottom, blue**) Type 3: in a Siemens whole-body acquisition (*CT WB 1.0 B30f*, 677 slices), the air gap between the patient’s thigh skin and clothing is misidentified as airway lumen (case *Dx-A0220_CT_WB_1_0_B30f_160816*).

Airway segmentation failures in the 33 excluded series cluster into three scanner-specific types (Figure 4). Manual inspection of all 306 series processed through VBP after airway segmentation revealed that 33 cases (10.8%) produced structurally implausible masks that could not be used for skeletonization or path planning. These failures arise from scanner-specific acquisition artifacts and weaknesses in the segmentation model itself. They cluster into three geometrically distinct types illustrated in Figure 4.

**Type 1—Bed center air channel (27 cases, 81.8%):** The segmentation model incorrectly labeled a narrow longitudinal air channel located beneath the patient table as the airway lumen (yellow circle, Figure 4, top row). This artifact is predominantly associated with **Philips** scanners: 26 of the 27 affected series record the manufacturer as *Philips* and the study description as *Chest 3D IMR* or *Chest 3D*, corresponding to the Philips Ingenuity TF PET/CT platform whose carbon-fiber couch contains a hollow longitudinal channel. The remaining case originates from a **GE Medical Systems** scanner (series, *Recon 2 5mm*, study: *chest.3d*), indicating that a geometrically similar bed structure is present in at least one GE platform as well. Because this structure presents as a continuous, low-attenuation (≈−1000 HU) tubular region at the inferior boundary of the CT field of view, it satisfies the same Hounsfield criteria as tracheal air.

**Type 2—Bed lateral edge (five cases, 15.2%):** The tapered lateral edge of the scanner bed produced a narrow wedge-shaped air pocket that was misclassified as a bronchial segment (red circle, Figure 4, middle row). This type spans two scanner platforms: three cases arise from **Siemens** whole-body acquisitions (series: *CT WB 3.0 B30f*; studies: *PET02/PET08WholebodyOnly Adult*; subjects A0164, A0216, A0221), and two cases arise from **Philips** chest acquisitions (series: *IMR* and *lung IDose iDose 4*; study: *Chest 3D IMR*; subject A0084). The mechanism is related to Type 1 but geometrically distinct: rather than the central channel, it is the sloped outer rim of the bed that creates the artifact.

**Type 3—Leg-skin–clothing air gap (one case, 3.0%):** In one Siemens whole-body case (*Lung_Dx-A0220*, series: *CT WB 1.0 B30f*; study: *PET08WholebodyOnly Adult*, 677 slices), the imaging field extends from the head to the lower extremities. When this volume is passed to a thorax-trained segmentation model, the low-attenuation gap between the patient’s thigh skin and clothing is misidentified as airway lumen (blue circle, Figure 4, bottom row), producing a skeleton that diverges entirely from the bronchial tree.

The root cause common to all three failure types is the limited generalizability of the airway segmentation model under real-world acquisition diversity.

Among the DICOM metadata fields available in the Lung-PET-CT-Dx dataset, *Manufacturer*, *SeriesDescription*, and *StudyDescription* are strongly predictive of Type 1, Type 2, and Type 3 failures, respectively. A DICOM-metadata-based pre-filter is a concrete near-term pipeline improvement.

## 5. Conclusions

We developed and validated **Virtual Bronchoscopic Pathfinder (VBP)**, a complete, open-source, web-based VBN system that integrates automated airway and tumor segmentation, topology-preserving skeletonization, multi-target bidirectional Dijkstra path planning with a three-tier anatomical cost field, and zero-footprint browser-based 3D visualization. Validated across 306 heterogeneous CT series from 154 lung cancer subjects, VBP achieved successful end-to-end path generation in 273 of 306 series (89.2%), with 33 cases (10.8%) excluded due to scanner-specific segmentation artifacts. Among the 273 anatomically valid cases, path generation succeeded in every instance. The three-tier cost field—a design not previously described in the VBN literature—provides a principled mechanism for tolerating skeleton discontinuities without pipeline failure. By building exclusively on open-source components and a public dataset, VBP demonstrates that reliable, end-to-end bronchoscopic navigation guidance can be delivered without proprietary hardware or software. The system is openly accessible at https://vbn.ziovision.ai (accessed on 21 February 2026) and serves as a scalable, reproducible foundation for imaging informatics research in bronchoscopic navigation, with a modular architecture that supports further extension toward intra-procedural guidance and multi-user clinical workflows.

## Figures and Tables

**Table 1:** Summary of the CT series retained from the Lung-PET-CT-Dx dataset after quality filtering.

Characteristic	Value
Source repository	Lung-PET-CT-Dx (TCIA) [22]; downloaded on 22 December 2025
Total series (after filtering)	306
Unique subjects	154
Adenocarcinoma (*Lung_Dx-A*)	116
Large-cell carcinoma (*Lung_Dx-B*)	9
Squamous cell carcinoma (*Lung_Dx-G*)	29
CT scanner manufacturer	Siemens 251 (82.0%), Philips 37 (12.1%), GE 18 (5.9%)
Acquisition period	2009–2011
Nominal slice thickness	1.0 mm (majority); 3–5 mm (minority)
Axial slices per series	200–852 (mean 346, median 350)
Reconstruction method	FBP 278 (90.8%), Iterative (iDose/IMR) 28 (9.2%)
Total axial images	105,931
Total data volume	≈54.6 GB

**Table 2:** Pipeline-level outcome summary across all 306 CT series.

Metric	Value
Total series processed	306 (154 subjects)
Segmentation failure (scanner artifact)	33 (10.8%)
Type 1: Bed centre air channel	27/33 (81.8%)
Type 2: Bed lateral edge	5/33 (15.2%)
Type 3: Leg skin–clothing gap	1/33 (3.0%)
Anatomically valid series	273 (89.2%)
Overall pipeline success (path generation)	273/306 (89.2%)

## Data Availability

The data presented in this study were derived from the Lung-PET-CT-Dx collection, available in the public domain through The Cancer Imaging Archive (TCIA) at https://doi.org/10.7937/TCIA.2020.NNC2-0461 (accessed/downloaded on 22 December 2025). The derived data supporting the findings of this study are available from the corresponding author upon reasonable request.

## Abbreviations

The following abbreviations are used in this manuscript:

VBP	Virtual Bronchoscopic Pathfinder
VBN	Virtual Bronchoscopic Navigation
PPL	Peripheral Pulmonary Lesion
CT	Computed Tomography
CAS	Connectivity-Aware Surrogate
LSD	Local-Sensitive Distance
COM	Center of Mass
LUT	Look-Up Table
TCIA	The Cancer Imaging Archive
SPA	Single-Page Application
FBP	Filtered Back Projection
HU	Hounsfield Unit
